# An online forum to support consultant psychiatrists in their first five years of practice, introduced during the COVID-19 pandemic

**DOI:** 10.1192/j.eurpsy.2021.1257

**Published:** 2021-08-13

**Authors:** T. Maclaren, N. Ahmed, S. Edwards

**Affiliations:** 1 Faculty Of Medicine, Imperial College London, London, United Kingdom; 2 Liaison Psychiatry, Central and North West London NHS Foundation Trust, London, United Kingdom; 3 Medical Staff, Central and North West London NHS Foundation Trust, London, United Kingdom

**Keywords:** wellbeing, Staff, Support, psychiatrists

## Abstract

**Introduction:**

In the United Kingdom, the move from trainee to consultant psychiatrist can be both exciting and daunting. Trainee psychiatrists have access to support and weekly supervision that is not available to consultants. Having an organised meeting for new consultants could help bridge this gap with peer-led support.

**Objectives:**

Improving support and guidance to new consultants Networking with peers Promoting wellbeing, good clinical practice and career development

**Methods:**

We identified a group of 85 consultants in their first five years of practice. Meetings were held online using videoconference. Senior leaders presented at each meeting, with a group discussion at the end. We surveyed attendees using an online platform.

**Results:**

We had excellent attendance rates from the group, with 30 to 45 consultants attending each webinar. Over 60% of attendees had been a consultant for less than a year. For 90%, this was their first experience of a new consultant forum. Attendees gave excellent feedback (Table). Being able to meet consultants from different specialties, hearing career stories from senior leaders and how they have managed the COVID-19 pandemic were cited as benefits.

**Table:** 

Table: Feedback scores (0 = not useful to 100 = very useful)	
Statement	Score
The forum helped me feel supported	75
Topics covered are relevant to me	79
I feel more connected with colleagues	71

**Conclusions:**

The forum was popular and the feedback was excellent. Using an online format worked well and made it easier to organise and plan sessions. There is potential to implement similar fora for other senior psychiatrists across Europe.

